# Emergency medical triage decisions are swayed by computer-manipulated cues of physical dominance in caller’s voice

**DOI:** 10.1038/srep30219

**Published:** 2016-07-26

**Authors:** Laurent Boidron, Karim Boudenia, Christophe Avena, Jean-Michel Boucheix, Jean-Julien Aucouturier

**Affiliations:** 1 Département de Médecine d’Urgence, Centre Hospitalier Universitaire, Dijon, France; 2 LEAD CNRS UMR5022, Université de Bourgogne, Dijon, France; 3 STMS CNRS UMR9912, IRCAM/CNRS/UPMC, Paris, France

## Abstract

In humans as well as other animals, displays of body strength such as power postures or deep masculine voices are associated with prevalence in conflicts of interest and facilitated access to resources. We conduct here an ecological and highly critical test of this hypothesis in a domain that, on first thought, would appear to be shielded from such influences: access to emergency medical care. Using acoustic manipulations of vocal masculinity, we systematically varied the perceived level of physical dominance of mock patients calling a medical call center simulator. Callers whose voice were perceived as indicative of physical dominance (i.e. those with low fundamental and formant frequency voices) obtained a higher grade of response, a higher evaluation of medical emergency and longer attention from physicians than callers with strictly identical medical needs whose voice signaled lower physical dominance. Strikingly, while the effect was important for physician participants, it was virtually non-existent when calls were processed by non-medically-trained phone operators. This finding demonstrates an unprecedented degree of vulnerability of telephone-based medical decisions to extra-medical factors carried by vocal cues, and shows that it may not simply be assumed that more medical training will shield decisions from such influences.

When making clinical decisions, physicians are advised to avoid non-medical contextual influences. The practice of medicine is conceived a sanctuary for rational and evidential thought, isolated from the passions of the world^1^. In reality, in a societal context favoring patient-centric attitudes and patients’ active participation in healthcare decisions, medical outcomes of the doctor-patient encounter are increasingly influenced by non-medical interactional factors^2^.

In many ways, the doctor-patient dyad is a power relationship. On the one hand, caregivers have decision-making power, privileged access to medical knowledge and are the gate-keepers to limited resources to which they can choose to refer patients, or not^3^,^4^. On the other hand, patients feel increasingly entitled to consumer rights, are better informed of possible diagnoses or treatment options, and consequently more demanding and pressuring^5^,^6^,^7^.

It is a delicate balance to maintain which, when done well, has positive associations to patient involvement and health outcomes^8^,^9^. But that balance can also go awry and negatively affect healthcare function and patient safety. Overly dominant physician behavior has been linked to reduced patient satisfaction^2^,^10^, reduced patient adhesion^11^ and malpractice claims^12^. Conversely, overly dominant patients may obtain inappropriate medication^13^,^14^,^15^ or referrals for unnecessary procedures^6^,^16^, which bear an important cost on the community and its capacity to care for other, more urgent cases.

When prompted in debriefing interviews to explain their giving in to patient pressure, physicians often invoke conscious strategic reasons, such as reducing patients’ anxiety with a “reassurance” referral^16^, or avoiding conflict with difficult or “celebrity” patients^17^. While the causal role of these variables on decision-making is rarely debated in the medical literature, a wealth of social psychological research in non-health contexts has shown that persuasion in truth often operates on the peripheral and automatic processing of individual-level traits, such as physical or vocal attractiveness^18^,^19^, status^20^, personality^21^ or physical dominance^22^, rather than on the supporting argumentation invoked to explain the outcome^20^,^23^,^24^. In the context of medicine, it is therefore possible that inappropriate outcomes of the doctor-patient interaction should result not only from the consideration of information specific to the situation, e.g. conceding antibiotics “strategically” to a particularly anxious or argumentative parent, but also on endogenous traits that are situation-independent, e.g. conceding a referral to a patient because he appears strong or attractive.

In this study, we use the vocal modality to elicit one of such cues (physical dominance), and test its influence on medical decision-making. Indeed, beyond a patient’s immediate visual appearance, his or her tone of voice, e.g. whether it is of a low or high pitch or whether its quality or timbre is deep or bright, can cue a listener about many traits of the speaker. Male speakers in particular have substantially lower fundamental frequency and narrower formant frequency dispersion than women^22^, a consequence of larger growth of vocal folds and tracts during male puberty^25^. Deeper masculine voices (i.e. those voices with lower fundamental frequency and narrower formant frequency dispersion) are perceived as belonging to males that are older^26^, larger^27^,^28^, more physically and socially dominant^29^ and more attractive to women^26^,^27^. Because vocal signals can be digitally altered to change both fundamental frequency and formant dispersion while keeping the associated verbal content constant^29^,^30^, manipulating the acoustical properties of patient speech recordings is therefore a powerful way to control for how doctors perceive traits such as physical dominance, independently of a given medical situation.

The specific medical context used in this study is that of telephone triage in emergency medical services (EMS). Triage is the process by which a patient telephone enquiry to an accident and emergency (A&E) department is assessed to determine the urgency of the problem and what resources should be dispatched^31^. Of all medical decision-making situations, EMS triage seems especially prone to extra-medical influences by vocal cues: dispatchers operate under uncertainty (because of incomplete sensory information), emergency and limited resources, while patients have high anxiety and expect an immediate response^32^,^33^. Here, we examined whether EMS dispatchers were influenced by the caller’s tone of voice to the point of dispatching more urgent and important resources to patients displaying increased physical dominance, regardless of their actual medical needs.

To do so, we designed a computer-based telephone simulator of a EMS triage center, in which real dispatchers (both physicians and non-medically-trained dispatch operators) received phone calls from virtual patients (VPs) with pre-recorded actor voices, and interacted with the callers via the invisible and real-time mediation of a human experimenter (Fig. 1). The behavior of the VP was controlled in real-time by the experimenter, who monitored every (unscripted) questions of the dispatcher and selected for each the most appropriate response in a set of about 80 pre-recorded sentences (e.g. about the problem’s history, symptoms, onset), which was then instantly played back to the dispatcher as in a natural interaction. Unbeknownst to both experimenter and dispatchers, we used acoustic manipulations of vocal masculinity (i.e. manipulated a speaker’s fundamental frequency and formant frequency dispersion) to systematically vary the perceived level of physical dominance of VP responses, and investigated whether dominant patients obtained better and more urgent responses, regardless of the medical information communicated to the dispatcher, which stayed constant across experimental conditions.

## Results

Acoustic transformations of vocal masculinity successfully manipulated the dispatchers’ perception of the caller’s physical dominance (M = 7.5 > M = 5.6; t(80) = 2.19, p = 0.03; Cohen’s d = 0.48). While female dispatchers evaluated the physical dominance of the caller more highly than male dispatchers (M = 7.5 > M = 5.6), there was no interaction of gender with the effect of the manipulation (F(1, 74) = 0.01, p = 0.91). Similarly, there was no interaction of the type of dispatcher (physician or operator) with the effect of the manipulation (F(1, 74) = 0.17, p = 0.68).

The transformations appeared to selectively manipulate physical dominance, rather than other related traits. In particular, the manipulation had no effect on either type of participants’ perception of caller’s age (M = 38.4 > M = 37.6, t(81) = 0.49, p = 0.61), social dominance (M = 8.3 > M = 7.7; t(81) = 0.88, p = 0.38) nor on the participants’ evaluation of a potential medicolegal risk with this case (M = 7.5 > M = 7.3; t(78) = 0.23, p = 0.81). Medicolegal risk was predicted by the VP’s social dominance for physician participants (*R*^2^ = 0.16, p = 0.02), but not for operators. The caller was deemed more likely to belong to higher (clerical: M = 6.3, lower managerial: M = 7.6, higher managerial: M = 6.6), than lower (agricultural: M = 2.6, long-term unemployed: M = 3.3, worker: M = 3.7) socio-economic categories (RP-ANOVA category: F(8, 616) = 30.8, p = 0.000), but the experimental condition did not interact with this distribution of category scores (RP-ANOVA conditionxcategory: F(8, 616) = 1.40, p = 0.19), and neither did the type of participants (RP-ANOVA conditionxcategoryxtype: F(8, 616) = 0.57, p = 0.79).

The two types of dispatchers reacted remarkably differently to the manipulation of caller’s dominance (Fig. 2). We encoded the priority level of the resources dispatched by the participants as a binary variable: high (Type-I and Type-II) and low (Type-III and Type-IV) priority. Physician dispatchers interacting with high-dominance VPs dispatched twice as many high-priority vehicles (88% of all their responses) as what they did for low-dominance VPs (41%; *χ*^2^ = 8.24, p = 0.004), whereas dispatch operators with no medical training had a similar rate of priority dispatches for high-dominance (29%) and non-dominance (25%) VPs (*χ*^2^ = 0.1, p = 0.75).

Physician dispatchers judged that cases presented by high-dominance VPs were of a higher degree of medical emergency than the (identical) cases presented by low-dominance VPs (M = 8.4 > M = 6.7; t(31) = 2.13, p = 0.04; Cohen’s d = 0.76), while operators made no such difference (M = 9.3 > M = 8.9, t(48) = 0.48, p = 0.62). In addition, physician participants spent more time interacting with high-dominance than low-dominance VPs (M = 104 > M = 73; t(32) = 2.47, p = 0.01; Cohen’s d = 0.87), whereas operators did not (M = 104 > M = 96; t(48) = 0.75, p = 0.45).

## Discussion

In short, callers whose voice were perceived as indicative of physical dominance (i.e. those whose voices had a lower frequency and a narrower format dispersion) obtained a higher grade of EMS response, a higher evaluation of medical emergency and longer attention from physicians than callers with strictly identical medical needs whose voice signaled lower physical dominance. The effect did not seem to result of a medicolegal strategy, as the experimental manipulation had no effect on the caller’s perceived social dominance or evaluation of medicolegal risk. Intriguingly, while the effect was important for physician participants, it was virtually non-existent for non-medically trained dispatch operators.

Because the acoustical properties of a voice inform the listener on the speaker’s physical traits, such as strength, age or size^29^, voice masculinity could in principle constitute factual evidence towards a valid medical diagnosis. However, it is unlikely that this was the case here: first, we did not manipulate the voice of the patient/child in need of assistance, but the voice of the caller/parent, whose physical status was irrelevant to the case. Second, if anything, increased physical dominance should prime robustness (or “phenotypic quality” – ref. 34), and not justify the dispatch of greater and more urgent resources. Finally, all information available to the participants in the scripted case unambiguously supported a non-emergency diagnosis. Even if the manipulation compelled physicians to spend more time interacting with high-dominance callers, the increase of collected information about the case should all the more so support a temperate, rather than excessive, dispatch decision. In the present situation, it therefore appears that physicians simply felt threatened or “bullied” into an irrelevant decision.

That physicians should more eagerly comply to the request for assistance of high, rather than low, physical dominance patients reinforces a much-studied pattern in both social and evolutionary psychology. In dyadic interactions, and most typically in structured work relationships^35^, interacting partners are found to “contrast on the control dimension”, i.e. to behave submissively when partners act dominantly^36^,^37^,^38^. Additionally, in most, if not all, human cultures, displays of body strength, as signaled by e.g. deep masculine voices^28^,^29^, are associated not only to reproductive^22^,^39^, but also cultural success such as facilitated access to resources^40^ and prevalence in conflicts of interest^41^. The importance of the present result lies in its demonstration of a large effect of voice masculinity in a field (medical decision-making) in which considerable normative strategies yet exist to avoid such extra-medical influences^42^ and in which such decisions have important consequences in terms of patient safety, medico-legal costs and availability of medical resources for other, more urgent cases^43^.

Only the physician participants, and not the non-medically trained dispatchers, were affected by the experimental manipulation. A large literature on expert performance^44^,^45^ would predict otherwise - namely, that medical training be linked to an increased ability to ignore extra-medical factors such as the ones manipulated here. While evidence exists that expert decisions are sometimes no more accurate than less-trained individuals’ decisions^46^,^47^, such situations remain confined to domains with weaker norms and less formal basis than emergency medicine, e.g. wine-tasting or violin-making^48^,^49^. It therefore does not seem appropriate to conclude here to a general lack of reliability of physicians’ decisions in telephone triage tasks. Physician participants’ increased sensitivity to vocal cues could even be taken to indicate, on the contrary, that they are more capable to integrate a variety of information, not only technical but para-technical, which is another known manifestation of expertise^44^. However, rather than differences linked to expertise, we instead suggest to look for an explanation in attitudinal differences towards the task of medical triage. Doctors do not only dispatch but, subsequently, give care. While a non-medically trained dispatch operator may never meet a calling patient again, and thus worry little about subsequent interaction, medical doctors involved in triage also likely anticipate subsequent medical care by themselves or a colleague. Such a personal stake in triage decisions likely comes with specific strategies or biases, e.g. towards favouring false-positive errors^50^ or maintaining good relationship with the patient^51^, that may explain here why doctors were more sensitive than non-doctors to the manipulation.

On the whole, this result has two important implications for healthcare organization in general, and telephone-based EMS in particular. First, it demonstrates an unprecedented degree of vulnerability of telephone-based medical decisions to extra-medical factors carried by vocal cues. Nonverbal vocal behavior is known to play a role in all medical interactions^10^,^12^ but, perhaps because it is the focus of a dispatcher’s exclusive attention in the case of EMS triage, care should be given to avoid situations where voice is conferred disproportionate persuasion power. The present effect of voice masculinity may even present a particular security issue as such acoustic manipulations can be produced in a low-cost, real-time and undetectable manner by many consumer digital audio processors^30^.

Second, the present result casts light on radical differences in the sensitivity of physicians and non-medical operators to the same vocal manipulations. While most industrial countries have adopted some form of telephone triage, national health policies differ in the type of personnel (A&E physicians, paramedics, nurses, phone operators, and combinations thereof) used to assess situations and dispatch resources, with a view to maintain balance between medical safety margins and an appropriate use of resources (see e.g. ref. 52 for the situation in France). The fact that operators with no medical training showed no sensitivity to the manipulated calls they had to process suggests that policy research needs to look not only into differences of assessment accuracy by medically and non-medically trained personnel^42^,^53^, but also to their differences in handling extra-medical influences, possibly mediated by different attitudes to the task of triage and subsequent care. In particular, it may not simply be assumed that more medical training will shield EMS dispatch decisions from such influences. The novel experimental paradigm presented here offers unprecedented flexibility to control medical information and to manipulate the non-verbal modality of its presentation, and its potential for such policy questions remains to be fully explored.

## Materials and Methods

### Participants

Thirty-four physicians and 50 triage operators (with no medical training) participated in the study, all experienced dispatchers in real-life EMS triage at the University Hospital of Dijon, France. Both groups had similar age (Physicians: M = 34.8, SD = 9.9; Operators: M = 37.7, SD = 8.7) and gender distributions (Physicians: 17 (50%) female (50%); Operators: 32 (64%) female). All participants had experience with the experimental set-up, which they had already used in at least one training session before the study. Five experimenters (male: 5) controlled the participants’ interaction with the VPs, all physicians experienced in EMS triage and in professional training of triage dispatchers. Participants and experimenters were randomly matched to one another, and to one of the two experimental conditions (high- vs low-dominance). All participants gave their written informed consent to participate in the study.

### Procedure

**Figure 1:**
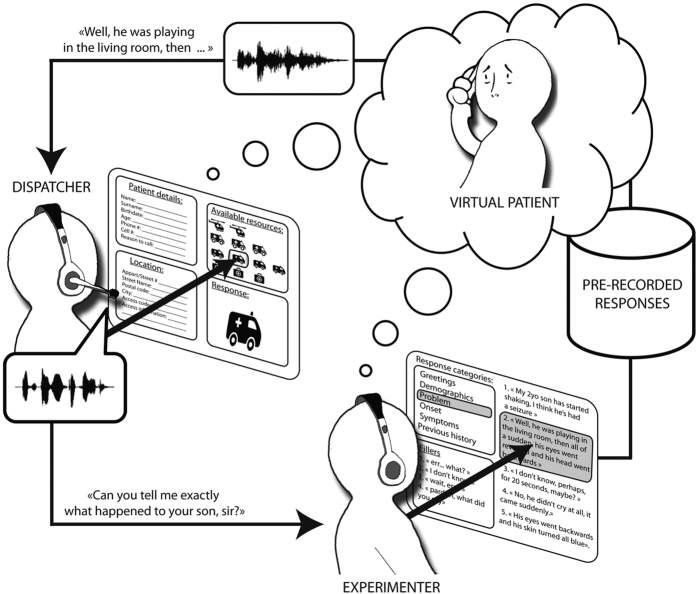
Physicians and dispatch operators received phone calls from virtual patients (VPs) with pre-recorded actor voices, and interacted with the callers via the invisible and real-time mediation of a human experimenter. The behavior of the VP was controlled in real-time by the experimenter, who monitored every (unscripted) questions of the dispatcher and selected for each the most appropriate response in a set of pre-recorded sentences.

**Figure 2:**
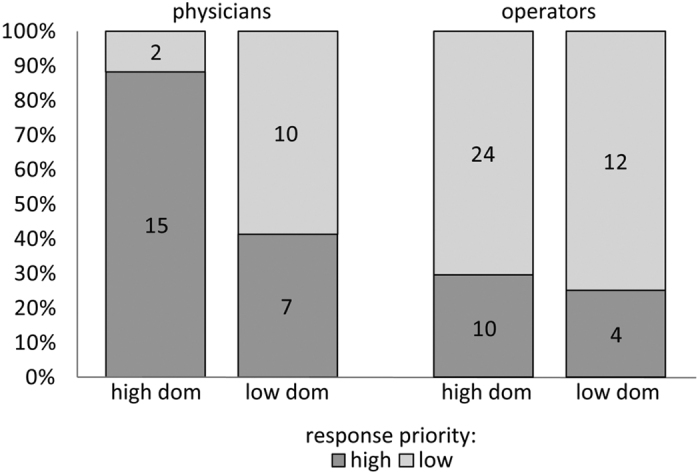
Without the experimenters’ and dispatchers’ knowing, we used acoustic manipulations of vocal masculinity to systematically vary the perceived level of physical dominance of VP responses. Physician dispatchers interacting with high-dominance VPs dispatched twice as many high-priority vehicles as those interacting with low-dominance VPs, while dispatch operators with no medical training had a similar rate of priority dispatches in both conditions.

All participants interacted with the same medical scenario (a parent calling for his convulsing 2-year-old child), presented in either a high-dominance or a low-dominance condition. Participants were shown to the simulator’s graphical interface, which reproduced a real-life EMS triage interface (Fig. 1). Participants were tasked to take an incoming call, and exercise the same protocol they would normally do in a real triage situation: ask and fill in patient details (demographics and location), take history about the problem (incl. onset, associated symptoms, timing, history of the same - and any other question deemed appropriate), decide on the most appropriate resource to dispatch, trigger the dispatch and close the call. Available resources corresponded to the typical array of resources available at the participants’ institution, and included four levels of emergency: Type-I (Emergency ambulance with advance life support; French: Unité mobile hospitalière), Type-II (Emergency ambulance with basic life support; French: Véhicule de secours aux asphyxiés et aux victimes), Type-III (Patient-transport ambulance; French: Ambulance agréée) and Type-IV (Home visit by a GP; French: Visite médicale). While participants interacted with the VP, and without their knowing, a human experimenter monitored the participants’ questions to the VP using a separate computer interface, coded them manually into question categories (e.g. demographics, problem onset), and selected the most appropriated VP response from a set of pre-recorded sentences corresponding to each question categories. Because their questions were interpreted in real-time by a human experimenter, dispatchers could have a natural, non-scripted conversation with the VP while each of their questions was accurately encoded for subsequent analysis. In addition, the system recorded the time and type of response dispatched by the participants as a consequence of their interaction with the VP. Finally, after the session, participants were asked to fill in a questionnaire with 10-point scales measuring their perception of the medical case (degree of medical emergency, degree of medico-legal risk) as well as the VP’s perceived social dominance (endpoints labeled “extremely dominant” and “extremely submissive” as defined in ref. 29), physical dominance (endpoints labeled “strongly agree” and “strongly disagree” below the statement: “If this man got in a fistfight with the average hospital worker, he would probably win”), likelihood to belong to each of a series of socio-economic categories (agricultural, employee, worker, clerical, lower managerial, higher managerial, own account workers, long-term unemployed, or retired; endpoints labeled “very likely” and “very unlikely”) and perceived age. This procedure was approved by the departmental ethics committee of the University of Bourgogne, and all methods were carried out in accordance with the approved guidelines.

### Medical scenario

All participants interacted with the same medical scenario, written by a physician experienced in EMS triage. The scenario described the case of a 2-year-old child, who just had hyperthermal convulsion. The caller is the father, calling from home, and the mother is also present. According to the father, the child has had cold symptoms and a moderate fever for 3 days. Before calling, the child started shaking and trembling, and his eyes were rolling, but fitting had stopped at the time of the call, and the child seemed well. The scenario was scripted to give the impression of a anxious father, with an assertive communication style, clear requests for resources being dispatched at his home, and a slightly annoyed attitude at having to negotiate them with the dispatcher. The scenario consisted of 78 short sentences (M = 4.3 s, max = 11 s), written to cover a variety of possible dispatcher questions and provide a sufficient amount of information about the case’s onset, timing, symptoms and history (see examples in SI_Audiofiles). The scenario was reviewed by 3 physicians trained in EMS dispatching, who all judged it umambiguously supported an etiology of hyperthermal convulsion, classified the case as “non-serious” and not requiring an emergency ambulance.

### Acoustical manipulations

Scripted responses from the VP were recorded by a male actor, and stored as uncompressed 16-bit, 48 kHz, ogg-format files. Two versions of the files were then produced to simulate a low and high physical dominance speaker, by manipulating the voice’s fundamental frequency and formant dispersion, two parameters related to vocal fold length and vocal tract length respectively^29^,^54^. In the low-dominance condition, the fundamental frequency of the recordings was increased by 1 semitone and formant dispersion decreased by 6%, giving the impression of a smaller, less masculine speaker. In the high-dominance condition, fundamental frequency was lowered by 1 semitone and formant dispersion increased by 6%, giving the impression of a larger and more masculine speaker. Both manipulations were produced without change in voice’s speech rate or tempo, using the Praat program^55^. From these two derivative sets of recordings, we formed a high-dominance scenario (with high-dominance manipulated recordings) and low-dominance scenario (with a low-dominance manipulated recordings). Because the manipulations only altered the acoustical characteristics of the voice, the two scenarios did not differ in terms of verbal content, VP communication style, and amount of medical information provided to the participants. Sample sound recordings from both conditions are available in SI.

## Additional Information

**How to cite this article**: Boidron, L. *et al*. Emergency medical triage decisions are swayed by computer-manipulated cues of physical dominance in caller’s voice. *Sci. Rep.* **6**, 30219; doi: 10.1038/srep30219 (2016).

## Supplementary Material

Supplementary S1 Audio

Supplementary S2 Audio

Supplementary S3 Audio

Supplementary S4 Audio

Supplementary S5 Audio

Supplementary S6 Audio

Supplementary S7 Audio

Supplementary S8 Audio

Supplementary S9 Audio

Supplementary S10 Audio

Supplementary Information
