# Supplementary material for: Emergency medical triage decisions are swayed by computer-manipulated cues of physical dominance in caller’s voice
Source: Sci Rep. 2016 Jul 26;6:30219. doi: 10.1038/srep30219 (PMC4960535; doi:10.1038/srep30219)
Supplement: Supplementary Information [file srep30219-s11.pdf]

Supporting information for

Emergency medical triage decisions are swayed by computer-manipulated cues of physical dominance in caller's voice

Laurent Boidron, Karim Boudenia, Christophe Avena, Jean-Michel Boucheix and Jean-Julien Aucouturier

Supporting information:

Audio item S1 : 14-.ogg: One of the 78 prerecorded answers of the script, processed for low-dominance condition (high pitch, high formant dispersion). Transcript: "*D'accord*" (okay)

Audio item S2 : 14+.ogg: Same recording as S1, processed for high-dominance condition (low pitch, small formant dispersion).

Audio item S3: 20-.ogg: One of the 78 prerecorded answers of the script, processed for low-dominance condition (high pitch, high formant dispersion). Transcript: "*Ah bin enfin le SAMU, bin c'est pas trop tôt, hein!*" (huh, someone at last, I nearly waited)

Audio item S4: 20+.ogg: Same recording as S3, processed for high-dominance condition (low pitch, small formant dispersion).

Audio item S5: 45-.ogg: One of the 78 prerecorded answers of the script, processed for low-dominance condition (high pitch, high formant dispersion). Transcript: "*Bin il jouait dans le salon et puis d'un seul coup euh, ses yeux se sont retournés, et sa tête est partie en arrière. Ensuite il est devenu tout bleu.*" (well, he was playing in the living room, then all of a sudden, his eyes rolled and his head shot backwards. Then his skin turned completely blue.)

Audio item S6 : 45+.ogg: Same recording as S5, processed for high-dominance condition (low pitch, small formant dispersion).

Audio item S7 : 59-.ogg: One of the 78 prerecorded answers of the script, processed for low-dominance condition (high pitch, high formant dispersion). Transcript: *"Alors d'ab- euh oui, il a un peu de temperature depuis trois jours environ 38"* (so firs- err yeah, he's got a bit of temperature the past 3 days, 38 celsius or so)

Audio item S8 : 59+.ogg: Same recording as S7, processed for high-dominance condition (low pitch, small formant dispersion).

Audio item S9 : 93-.ogg: One of the 78 prerecorded answers of the script, processed for low-dominance condition (high pitch, high formant dispersion). Transcript: *"Un docteur? Il ne va... Mais il va arriver dans les combien de temps? Et si ça recommence, et qu'est ce que je fais?!"* (Sending me a GP? But he… but how long till he makes it here? And what if he does it again, what am I supposed to do then?!)

Audio item S10 : 93+.ogg: Same recording as S9, processed for high-dominance condition (low pitch, small formant dispersion).
